# Evaluation of a Solid Dispersion of Curcumin With Polyvinylpyrrolidone and Boric Acid Against *Salmonella* Enteritidis Infection and Intestinal Permeability in Broiler Chickens: A Pilot Study

**DOI:** 10.3389/fmicb.2018.01289

**Published:** 2018-06-15

**Authors:** Daniel Hernandez-Patlan, Bruno Solis-Cruz, Karine Patrin Pontin, Juan D. Latorre, Mikayla F. A. Baxter, Xochitl Hernandez-Velasco, Ruben Merino-Guzman, Abraham Méndez-Albores, Billy M. Hargis, Raquel Lopez-Arellano, Guillermo Tellez

**Affiliations:** ^1^Laboratorio 5: LEDEFAR, Unidad de Investigacion Multidisciplinaria, Facultad de Estudios Superiores Cuautitlan, Universidad Nacional Autonoma de México, Cuautitlan Izcalli, Mexico; ^2^Departamento de Medicina Veterinária Preventiva, Centro de Diagnóstico e Pesquisa em Patologia Aviária, Universidade Federal do Rio Grande do Sul, Porto Alegre, Brazil; ^3^Department of Poultry Science, University of Arkansas, Fayetteville, AR, United States; ^4^Departamento de Medicina y Zootecnia de Aves, Facultad de Medicina Veterinaria y Zootecnia, Universidad Nacional Autónoma de México, Mexico City, Mexico; ^5^Laboratorio 14: Alimentos, Micotoxinas y Micotoxicosis, Unidad de Investigacion Multidisciplinaria, Facultad de Estudios Superiores Cuautitlan, Universidad Nacional Autonoma de México, Cuautitlan Izcalli, Mexico

**Keywords:** boric acid, chickens, curcumin, polyvinylpyrrolidone, *Salmonella*

## Abstract

In the present study, *in vitro* assays were conducted to evaluate the solubility of curcumin (CUR) alone or with polyvinylpyrrolidone (PVP) at different pH, as well as its permeability in Caco-2 cells. Results confirmed that the solid dispersion of CUR with PVP (CUR/PVP) at a 1:9 ratio, significantly increased (*P* < 0.05) solubility and permeability compared to CUR alone. Then, the antimicrobial activity of CUR/PVP, boric acid (BA), and a combination of 0.5% CUR/PVP and 0.5% BA (CUR/PVP-BA) against *Salmonella* Enteritidis (SE) was determined using an *in vitro* digestion model that simulates crop, proventriculus, and intestine. The results revealed that in the proventriculus and intestinal compartments significant reductions of SE were observed in all the experimental treatments, but 1% BA eliminated SE in the intestinal compartment and CUR/PVP-BA showed a synergistic effect on antimicrobial activity against SE. To complement these findings, two independent *in vivo* trials were conducted to determine the effect of 0.1% CUR/PVP; 0.1% BA; or the combination of 0.05% CUR/PVP (1:9 ratio) and 0.05% BA (CUR/PVP-BA) on the antimicrobial activity against SE, intestinal permeability and inflammatory responses in broiler chickens. BA at 0.1% had no significant *in vivo* effects against SE. However, the combination of 0.05% BA and 0.05% CUR/PVP and 0.05% BA was sufficient to reduce crop and intestinal SE colonization in broiler chickens in two independent trials, confirming the synergic effect between them. A similar antimicrobial impact against SE intestinal colonization was observed in chickens treated with 0.1% CUR/PVP at a 1:9 ratio, which could be due to the increase in solubility of CUR by PVP. Furthermore, 0.1% CUR/PVP reduced the intestinal permeability of FITC-d and total intestinal IgA, as well as increase the activity of SOD when compared to control, while, CUR/PVP-BA only decreased SOD activity. Further studies to confirm and expand the *in vivo* results obtained in this pilot study, adding intestinal microbial commensal groups and more inflammatory biomarkers to get a complete description of the effects of BA and CUR deserves further investigation.

## Introduction

*Salmonella enterica* serovar Enteritidis is a pathogen frequently found in the gastrointestinal tract of poultry which can also cause foodborne illness in humans. The primary route of transmission from animals to humans is through contaminated food or foodstuffs such as eggs, egg products and poultry meat (Mani-López et al., 2012; Bertelloni et al., 2017). It is estimated that the consequences of foodborne diseases are substantial (WHO, 2018). Furthermore, the use of antibiotics as growth promoters in animal production have stressed the need for alternatives that can offer similar performance benefits. These alternatives include probiotics, prebiotics and plant extracts (Huyghebaert et al., 2011); bacteriophages (Andreatti Filho et al., 2007); and organic acids (Menconi et al., 2011). Boron is an essential nutrient for animals, plants, and fungi.

Boron is a weak acid pKa = 9.1) and, at physiological pH, is present mostly as BA (H_3_BO_3_) (Hunt, 1994, 2003). BA is used as antibacterial, pesticide, fire retardant, and a precursor to other chemicals (Kabu and Akosman, 2013). On the other hand, CUR, is a curcuminoid of turmeric (*Curcuma longa*), a member of the ginger family, *Zingiberaceae*, used as an herbal food supplement, cosmetic and food coloring. CUR is a diarylheptanoid (natural phenols) with antioxidant and anti-inflammatory properties (Bereswill et al., 2010; Wanninger et al., 2015). In poultry production, CUR has been used as a growth promoter (Khan et al., 2012; Rajput et al., 2013) and antimicrobial (Lawhavinit et al., 2010; Moghadamtousi et al., 2014) compound. In a recent *in vitro* study published by our laboratories, we showed that 1% CUR, had no antimicrobial effect against SE; however, the combination of BA and CUR have a synergistic bactericidal impact against SE (Hernandez-Patlan et al., 2017). However, one major limitation of CUR is its low solubility and bioavailability. PVP is a water-soluble synthetic polymer used as a binder and as a lubricant for eye drops (Haaf et al., 1985). Furthermore, it has been reported that PVP can improve the solubility and dissolution of many poorly water-soluble drugs as CUR (Kaewnopparat et al., 2009).

The objectives of the present study were, first to evaluate the solubility and permeability of CUR combined with PVP *in vitro.* Second, to evaluate the *in vitro* antimicrobial effect of a solid CUR/PVP at a 1:9 ratio, BA and the combination of 0.5% CUR/PVP and 0.5% BA (CUR/PVP-BA) against SE since some interactions between them have been reported. Third, to evaluate the *in vivo* effects of these feed additive candidates on SE colonization to confirm the interactions between CUR/PVP and BA, and their effects in some inflammation parameters in broiler chickens.

## Materials and Methods

### Solubility Studies

The solubility of CUR was assessed by adding an excess amount of CUR (200 mg) (≈50%, Mixim Laboratories, Naucalpan, Mexico) to 20 mL of aqueous buffer solutions at pH 1.2, 5.2, and 6.4, containing or not PVP (Agrimer K-30, Ashland, Columbus, OH, United States) in 1:6 and 1:9 ratios (CUR/PVP). The suspensions formed were equilibrated under continuous agitation for 24 h at room temperature. Then centrifuged (Beckman Coulter Microfuge 20R, United States) at 2292 × *g* for 10 min at 4°C and the supernatants were filtered through a 0.45 μm pore size Millipore membrane filter. The filtrates were analyzed with a UV/VIS spectrophotometer (Varian Cary 100, United States) at 421 nm. Solubility studies were performed in triplicate. The concentration of soluble CUR was determined using a calibration curve with a concentration range of 1.36–13.6 μg/mL.

### Caco-2 Cell Culture

The Caco-2 human colon cancer cells from the American Type Culture Collection (ATCC, Rockville, MD, United States) at passage 16 were cultured in T-75 cm^2^ flasks containing Dulbecco’s modified eagle medium (DMEM, Gibco-Invitrogen, Thermo Fisher Scientific, Illkirch-Graffenstaden, France) supplemented with glucose (4.5 g/L), FBS (15%; v/v), vitamins (2%; v/v), nonessential amino acid solution (2%; v/v), L-glutamine (2%; v/v), and antibiotic solution (2%; v/v), at 37°C in a humidified atmosphere of 5% CO_2_/95% air. When the cell culture reached 80–90% confluence, cells were dispersed with 0.025 M trypsin-EDTA and reseeded in a new flask. Medium was replaced every 3 days.

### Permeability Studies

Caco-2 cells were seeded on the apical side of Transwell^®^ permeable supports (Transwell Corning^®^, Kennebunk, EU, 0.4 μm polycarbonate membrane, 1.12 cm^2^ surface and a volume of 0.5 mL in the apical compartment and 1.5 mL in the basolateral compartment) at a density of 2 × 10^4^ cells per well for 21 days. The culture medium was changed every 3 days.

Permeability studies were performed after complete cell differentiation and only wells that exhibited transepithelial electrical resistance (TEER, MERSSTX01 electrode, Millicell ERS-2, Millipore, Billerica, MA, United States) greater than 600 Ω cm^2^ were used. Suspensions of CUR alone or with PVP in 1:6 and 1:9 ratios were diluted in DMEM to reach a concentration of 250 μg CUR/mL (Equivalent to 125 μg of CUR per well). Before starting the experiment, the culture medium was removed, and cell monolayers were washed twice with 1 mL of PBS buffer. Then, 0.5 mL of each diluted CUR suspension was added to the apical side of the cell monolayers, while on the basolateral side 1.5 mL of DMEM was placed. Cells were incubated at 37°C for 120 min. Passage of CUR to the basolateral side was determined by UPLC-TQ-ESI-MS/MS (Waters ACQUITY UPLC system, Milford, MA, United States). Chromatographic analysis was done using a Waters ACQUITY BEH Shield RP 18 column (2.1 mm × 100 mm, 1.7 μm). The mobile phase was composed of 60% A (acetonitrile and 0.1% formic acid) and 40% B (0.1% formic acid solution). The flow rate of the mobile phase was 0.25 mL/min, and the injection volume was 7.5 μL. The column temperature was conditioned at 30°C, and the autosampler was maintained at 4°C.

Results are expressed as apparent permeability coefficient (Papp) and absorption enhancement ratio (R), according to the following equations:

$$\mathrm{Papp}\;(cm/s)=\left(\frac{\mathrm{dQ}}{\mathrm{dt}}\right)\times \left(\frac{1}{A\times C_{0}}\right)$$

Where *dQ*/*dt* is the steady-state flux, *A* is the surface area of the filter and *C*_0_ is the initial concentration in the donor chamber (Apical side).

$$R=\frac{P_{\mathrm{app}}\mathrm{Test}}{P_{\mathrm{app}}\mathrm{Ctrl}}$$

Where PappCtrl is the average of the apparent permeability coefficient of CUR alone suspended in DMEM and PappTest is the average of the apparent permeability coefficient of CUR/PVP suspensions in 1:6 and 1:9 ratios.

### Bacterial Strain and Culture Conditions

The organism used was a poultry isolate of *Salmonella enterica* serovar Enteritidis (SE), bacteriophage type 13A, obtained from the USDA National Veterinary Services Laboratory (Ames, IA, United States). The isolate is resistant to 25 μg/mL of novobiocin (NO, catalog no.N-1628, Sigma) and was selected for resistance to 20 μg/mL of nalidixic acid (NA, catalog no.N-4382, Sigma) in our laboratory. In this study, 100 μL of SE from a frozen aliquot was added to 10 mL of tryptic soy broth (Catalog No. 22092, Sigma) and incubated at 37°C for 8 h, and passed three times every 8 h to ensure that all bacteria were in log phase as previously described (Lin et al., 1995). Post-incubation, bacterial cells were washed three times with sterile 0.9% saline by centrifugation at 1864 × *g* for 10 min, reconstituted in saline, quantified by densitometry with a spectrophotometer (Spectronic 20D+, Spectronic Instruments Thermo Scientific, Rochester, NY, United States), and diluted to an approximate concentration of 4 × 10^7^ cfu/mL. Concentrations of SE were further verified by serial dilution and plated on brilliant green agar (BGA, Catalog No. 70134, Sigma) with NO and NA for enumeration of actual cfu used in the experiment.

### Preparation of Treatments and Diets

#### *In Vitro* Experiments

The treatments for the *in vitro* experiments were: (1) a control non-supplemented diet; (2) basal diet plus 1% BA (99–100%, USP grade, Drogueria Cosmopolita, Naucalpan, Mexico City, Mexico); (3) basal diet plus 1% CUR/PVP; and (4) basal diet plus a BA-CUR/PVP.

#### *In Vivo* Experiments

In the case of the *in vivo* experiments the treatments were: (1) a control non-supplemented diet; (2) basal diet plus 0.1% BA; (3) basal diet plus 0.1% CUR/PVP; and 4) basal diet plus BA-CUR/PVP.

In all the treatments, the particle size was homogenized using a No. 25 mesh sieve to obtain particles of around 700 μm. In both *in vitro* and *in vivo* experiments, treatment 3 was prepared by dissolving 10 g of CUR in a solution containing 90 g of PVP (CUR/PVP in a 1: 9 ratio) followed by the solvent evaporation at 40°C for 48 h and subsequently sieving. For treatment 4, in both *in vitro* and *in vivo* experiments, 50 g of BA and 50 g of CUR/PVP were combined, granulated, dried and sieved. Once prepared, each of the treatments was added to the feed in a concentration of 1% (10 g/kg of feed) to obtain the diets used in the *in vitro* studies and 0.1% (1 g/kg of feed) for *in vivo* studies. Starter feed used in this experiment was formulated to approximate the nutritional requirements for broiler chickens as recommended by the National Research Council (1994), and adjusted to breeder’s recommendations (Cobb-Vantress, Inc., 2015). No antibiotics, coccidiostats, or enzymes were added to the feed (**Table 1**).

**Table 1:** Ingredient composition and nutrient content of a basal starter diet used for both *in vitro^1^* and *in vivo^2^* studies.

Item	Corn soybean-based diet
Ingredients (g/kg)	
Corn	574.5
Soybean meal	346.6
Poultry oil	34.5
Dicalcium phosphate	18.6
Calcium carbonate	9.9
Salt	3.8
DL-Methionine	3.3
L-Lysine HCL	3.1
Threonine	1.2
Choline chloride 60%	2.0
Vitamin premix^3^	1.0
Mineral premix^4^	1.0
Antioxidant^5^	0.5
Calculated analysis	
Metabolizable energy (MJ/kg)	12.7
Crude protein (g/kg)	221.5

*^*1*^For in vitro studies, each of the treatments was added to the feed in a concentration of 1% (10 g/kg of feed). ^*2*^For in vivo studies, each of the treatments was added to the feed in a concentration of 0.1% (1 g/kg of feed). ^*3*^Vitamin premix supplied per kg of diet: Retinol, 6 mg; cholecalciferol, 150 μg; dl-α-tocopherol, 67.5 mg; menadione, 9 mg; thiamine, 3 mg; riboflavin, 12 mg; pantothenic acid, 18 mg; niacin, 60 mg; pyridoxine, 5 mg; folic acid, 2 mg; biotin, 0.3 mg; cyanocobalamin, 0.4 mg. ^*4*^Mineral premix supplied per kg of diet: Mn, 120 mg; Zn, 100 mg; Fe, 120 mg; copper, 10–15 mg; iodine, 0.7 mg; selenium, 0.2 mg; and cobalt, 0.2 mg. ^*5*^Ethoxyquin.*

### Determination of Antimicrobial Activity Using an *in Vitro* Digestion Model

An *in vitro* digestive assay previously described (Latorre et al., 2015; Hernandez-Patlan et al., 2017) was used to determine the antimicrobial activity of CUR/PVP in a 1:9 ratio, BA and CUR/PVP-BA at 1% (w/w). Experiments were run in triplicate. Briefly, the digestive model used allows simulating three main compartments of the chicken gastrointestinal tract. The first gastrointestinal compartment simulated was the crop, where 5 g of feed and 10 mL of 0.03 M hydrochloric acid (HCl; EMD Millipore Corporation, Billerica, MA, United States) were placed in 50 mL polypropylene centrifuge tubes, reaching a pH value around 5.2. In this compartment, 1 mL of SE (10^8^ cfu/mL) was added. Tubes were then incubated for 30 min and mixed with a standard orbital shaker (19 rpm; VWR). To simulate the proventriculus as the next gastrointestinal compartment, 3000 U of pepsin per g of feed (Sigma-Aldrich, St Louis, MO, United States) and 2.5 mL of 1.5 M HCl was added to each tube, reaching a pH of 1.4–2.0. Tubes were incubated for additional 45 min. The third and final step was intended to simulate the intestinal section of the gastrointestinal tract. For that, 6.84 mg of 8× pancreatin (Sigma-Aldrich) in 6.5 mL of 1.0 M sodium bicarbonate (Sigma-Aldrich) was added, reaching a pH value between 6.4 and 6.8. Samples were further incubated for 2 h (total incubation time 3.25 h). In each compartment, a sample of 1 mL was taken and centrifuged for 30 min at 1864 × *g*. After that, the supernatants were discarded, and the pellets were reconstituted with 988 μL of 0.9% sterile saline and 12 μL of PrestoBlue^®^. These samples were incubated for 60 min at 40°C and centrifuged again for 30 min at 1864 × *g*. Finally, the supernatants were recovered and a sample of 100 μL was taken and placed in 96-well flat bottom Bacti plates (Catalog No. 269787; Nalgene Nunc International, Rochester, NY, United States) to measure the fluorescence intensity, which was measured a at an excitation wavelength of 530 nm and an emission wavelength of 590 nm (Synergy HT, multimode microplate reader; BioTek Instruments, Inc., Winooski, VT, United States). The fluorescence units of the samples were converted to concentration and reported as cfu/mL of SE using a calibration curve as previously described (Hernandez-Patlan et al., 2017).

### Animal Source, Diets, and Experimental Design

In the present study, two independent trials were conducted. In each experiment, 60 day-of-hatch male Cobb-Vantress broiler chickens (Fayetteville, AR, United States) were randomly allocated to one of four groups (*n* = 15 chickens). Chicks were housed in brooder battery cages, provided with their respective diet and water *ad libitum*, and maintained at an age-appropriate temperature during the 7 days of the experiments. All animal handling procedures complied with Institutional Animal Care and Use Committee (IACUC) at the University of Arkansas, Fayetteville (protocol #15006). At day 6, all chicks were orally challenged with 10^7^ cfu of SE per bird. The concentration of FITC-d was calculated based on group body weight; therefore, groups were weighed at 6 d of age. All chickens were given an appropriate dose of FITC-d by oral gavage at 7 days of age. One-hour post-FITC-d gavage, all chickens were euthanized by CO_2_ inhalation, and blood samples were collected from the femoral vein and centrifuged (1000 × *g* for 15 min) to separate the serum from the red blood cells for FITC-d and SOD determination. Furthermore, intestinal samples for total IgA levels, as well as crop and CCT to evaluate SE recovery were also collected.

### *Salmonella* Recovery

In this experiment, crop and CCT were homogenized and diluted with saline (1:4 by w/v), and 10-fold dilutions were plated on BGA with NO and NA, incubated at 37°C for 24 h to enumerate total SE colony forming units. Subsequently, the crop and CCT samples were enriched in 2× concentrated tetrathionate enrichment broth and further incubated at 37°C for 24 h. Following this, enrichment samples were confirmed negative for *Salmonella* by streak plating on Xylose Lysine Tergitol-4 (XLT-4, Catalog No. 223410, BD Difco^TM^) selective media.

### Serum Determination of FITC-d Leakage

Intestinal leakage of FITC-d (MW 3–5 kDa; Sigma-Aldrich Co., St. Louis, MO, United States) and the measurement of its serum concentration were determined since FITC-d is a marker of paracellular transport and mucosal barrier dysfunction (Yan et al., 2009; Kuttappan et al., 2015; Vicuña et al., 2015a,b). One hour before the chicks were humanely killed by CO_2_ inhalation, 12 chicks of each group were given an oral gavage dose of FITC-d (8.32 mg/kg), and the rest of chickens were used as controls. FITC-d levels of diluted sera were measured at excitation wavelength of 485 nm and an emission wavelength of 528 nm (Synergy HT, Multi-mode microplate reader, BioTek Instruments, Inc., VT, United States) (Baxter et al., 2017).

### Superoxide Dismutase Activity

Superoxide dismutase activity was measured in serum samples using a commercial assay kit (Cayman chemical company, Item No. 706002, Ann Arbor, MI, United States) following the manufacturer’s instructions. Three types of SOD (Cu/Zn, Mn, and FeSOD) were determined and the optimal dilution to quantify the SOD activity was 1:5. Samples were measured at 450 nm using an ELISA plate reader (Synergy HT, multi-mode microplate reader, BioTek Instruments, Inc., Winooski, VT, United States).

### Intestinal Total Immunoglobulin A (IgA) Levels

Total IgA levels were determined in gut rinse samples as previously described (Merino-Guzmán et al., 2017). Briefly, an intestinal section of 5 cm from Meckel’s diverticulum was taken and rinsed three times with 5 mL 0.9% saline; then the rinse was collected in a tube and centrifuged at 1864 × *g* at 4°C for 10 min. The supernatant was poured into a 96-microwell plate and stored at -20°C until tested. A commercial indirect ELISA set was used to quantify IgA according to the manufacturer’s instructions (Catalog No. E30-103, Bethyl Laboratories, Inc., Montgomery, TX, United States). 96-well plates (Catalog No. 439454, Nunc MaxiSorp, Thermo Fisher Scientific, Rochester, NY) were used, and samples were measured at 450 nm using an ELISA plate reader (Synergy HT, multi-mode microplate reader, BioTek Instruments, Inc., Winooski, VT, United States). The obtained chicken IgA concentration was multiplied by the dilution factor (1:100) to determine the amount of chicken IgA in the undiluted samples.

### Statistical Analysis

Data from bacterial counts (SE Log_10_ cfu/g) in crop and CCT, SOD activity, total IgA levels and serum FITC-d concentration were subjected to analysis of variance as a completely randomized design, using the General Linear Models procedure of SAS (SAS Institute, 2002). Significant differences among the means were determined by Duncan’s multiple-range test at *P* < 0.05. Enrichment data were expressed as positive/total chickens (%), and the percent recovery of SE was compared by chi-squared analysis (Zar, 1984), testing all possible combinations to determine the significance (*P* < 0.05).

## Results

The results of the solubility studies of CUR alone or with PVP at different ratios and pH conditions under room temperature are summarized in **Table 2**. At pH 1.2 and 5.2, CUR showed a very low solubility, and at pH 6.4 the solubility was not determined due to CUR degradation. However, when CUR was mixed with PVP, the solubility of CUR was significantly improved (*P* < 0.05), particularly at a CUR-PVP ratio of 1:9.

**Table 2:** Solubility of curcumin (CUR) alone or with polyvinylpyrrolidone (PVP) in different proportions at pH 1.2, 5.2, and 6.4 under room temperature^1^.

Formulation	CUR Concentration (μg/mL)		
	pH = 1.2	pH = 5.2	pH = 6.4
CUR	<1.36^c^	<1.36^c^	ND^∗^
CUR/PVP (1:6)	39.95 ± 0.98^b^	85.57 ± 2.02^b^	94.27 ± 4.70^b^
CUR/PVP (1:9)	122.06 ± 2.59^a^	184.04 ± 5.56^a^	212.82 ± 1.20^a^

*^*1*^Data are expressed as mean ± SE. ^*a*-*c*^ Values within columns with different superscripts differ significantly (P* < *0.05); n = 3. ^∗^Not determined due to instability of CUR (degradation)*.

**Table 3** shows the results of the evaluation of mean apparent permeability (Papp) and the absorption enhancement ratio (R) of CUR with PVP at different ratios across Caco-2 cell monolayers after two h incubation. The permeability of CUR in the Caco-2 cells was significantly improved by CUR-PVP ratio 1:9, followed by CUR-PVP ratio 1:6. The lowest permeability value was for CUR alone (**Table 3**).

**Table 3:** Evaluation of mean apparent permeability (Papp) and the absorption enhancement ratio (R) of curcumin (CUR) with polyvinylpyrrolidone (PVP) at different ratios across Caco-2 cell monolayers after 2 h incubation^1^.

Formulation	Absorption		P_APP_ × 10^-6^ (cm/s)	*R*
	μg	%		
CUR	10.00 ± 0.42^c^	8	4.96 ± 0.21^c^	–
CUR/PVP (1:6)	21.44 ± 3.42^b^	17.16	10.64 ± 1.69^b^	2.14
CUR/PVP (1:9)	66.00 ± 13.58^a^	52.80	32.74 ± 6.74^a^	6.60

*^*1*^Data are expressed as mean ± SE. ^*a*-*c*^ Values within columns with different superscripts differ significantly (P* < *0.05); n = 3*.

The results of the evaluation of the antimicrobial activity of CUR/PVP, BA or CUR/PVP-BA on SE using the *in vitro* digestion model and a fluorometric method with PrestoBlue^®^ are summarized in **Table 4**. None of the experimental groups reduced SE in the crop compartment. However, significant reductions in SE were observed in all the experimental treatments in the compartments that simulate the proventriculus and intestine. It was remarkable to observe that in just 3.25 h, 1% BA eliminated SE in the intestinal compartment (**Table 4**).

**Table 4:** Evaluation of the antimicrobial activity of curcumin with polyvinylpyrrolidone (CUR/PVP, 1:9 ratio), boric acid (BA), and CUR/PVP-BA^1^ on *Salmonella* Enteritidis (SE)^2^ using an *in vitro* digestion model and a fluorometric method with PrestoBlue^®^^3^.

Treatments	Compartment		
	Crop	Proventriculus	Intestine
Control	7.78 ± 0.00^a^	5.03 ± 0.12^a^	7.23 ± 0.00^a^
1% BA	7.78 ± 0.00^a^	3.81 ± 0.05^b^	0.00± 0.00^c^
1% CUR/PVP	7.73 ± 0.00^a^	3.91 ± 0.08^b^	5.00 ± 0.55^b^
0.5% BA + 0.5% CUR/PVP	7.65 ± 0.00^ab^	3.71 ± 0.05^b^	3.94 ± 0.23^b^

*^*1*^Each mean is represented by three observations (n = 3) ± SE. ^*2*^Inoculum used 10^*8*^ cfu/mL of SE. ^*3*^Data are presented in Log_*10*_ cfu/mL. ^*a*-*c*^Values within treatment columns for each treatment with different superscripts differ significantly (P* < *0.05)*.

**Table 5** summarizes the *in vivo* results of CUR/PVP, BA or CUR/PVP-BA administration on SE colonization in the crop and CCT of broiler chickens in trial 1 and trial 2. There was no reduction of SE colonization in the crop in chicks receiving 0.1% CUR/PVP or 0.1% BA in both trials. However, when products were used in combination, there was a significant reduction in SE colonization in the crop, which was consistent in both trials. This synergistic effect was even more apparent after enrichment of the samples. Chickens receiving 0.1% of CUR/PVP or 0.1% CUR/PVP-BA (0.05% CUR/PVP and 0.05% BA) had a significant reduction in the number of positive SE samples in the crop and CCT compared to the control non-treated chickens (**Table 5**). The results of the dietary administration of CUR/PVP, BA or CUR/PVP-BA on serum FITC-d concentration, SOD activity and IgA levels in broiler chickens are shown in **Table 6**. In the present study, no significant difference was observed in body weight and body weight gain between treatments (Data not shown).

**Table 5:** Evaluation of curcumin with polyvinylpyrrolidone (CUR/PVP, 1:9 ratio), boric acid (BA), and CUR/PVP-BA on crop and ceca-cecal tonsils (CCT) colonization of *Salmonella* Enteritidis (SE)^1^ in broiler chickens in trial 1 and trial 2^2^.

Treatments	Crop Log_10_ cfu/g	Crop +/- (%)^3^	CCT Log_10_ cfu/g	CCT +/- (%)^3^
	**Trial1**			
Control	2.68 ± 0.47^ab^	9/12 (75%)	4.01 ± 0.29^a^	12/12 (100%)
0.1% BA	2.79 ± 0.63^ab^	8/12 (67%)	2.91 ± 0.40^ab^	10/12 (83%)
0.1% CUR/ PVP	3.08 ± 0.57^a^	9/12 (75%)	2.42 ± 0.54^b^	8/12 (67%)^∗^
0.05% BA + 0.05% CUR/PVP	1.21 ± 0.52^b^	4/12 (33%)^∗^	2.39 ± 0.52^b^	8/12 (67%)^∗^

	**Trial2**			
Control	2.69 ± 0.48^a^	9/12 (75%)	3.94 ± 0.22^a^	12/12 (100%)
0.1% BA	2.72 ± 0.70^a^	8/12 (67%)	2.74 ± 0.49^abc^	9/12 (75%)
0.1% CUR/ PVP	3.19 ± 0.47^a^	9/12 (75%)	2.34 ± 0.50^c^	8/12 (67%)^∗^
0.05% BA + 0.05% CUR/PVP	0.93 ± 0.49^b^	3/12 (25%)^∗∗^	2.47 ± 0.54^bc^	8/12 (67%)^∗^

*^*1*^Data are presented in Log_*10*_ cfu /g of tissue. Mean ± SE from 12 chickens. ^*a*-*c*^Values within treatment columns for each treatment with different superscripts differ significantly (P < 0.05). ^*2*^Chickens were orally gavaged with 10^*7*^ cfu of SE per chicken at 6-day old, samples were collected 24 h later. ^*3*^Data are presented as positive/total chickens (%). ^∗^P < 0.05; ^∗∗^P < 0.01*.

**Table 6:** Evaluation of curcumin with polyvinylpyrrolidone (CUR/PVP, 1:9 ratio), boric acid (BA), and CUR/PVP-BA on serum concentration of fluorescein isothiocyanate–dextran (FITC-d), superoxide dismutase (SOD) activity and immunoglobulin A (IgA) levels in broiler chickens^1^.

Treatments	FITC-d (μg/mL)	SOD (U/mL)	IgA (μg/mL)
Control	0.591 ± 0.055^a^	3.58 ± 0.31^b^	14.21 ± 0.83^a^
0.1% BA	0.534 ± 0.046^ab^	3.58 ± 0.25^b^	12.35 ± 0.78^ab^
0.1% CUR/ PVP	0.432 ± 0.037^b^	4.48 ± 0.20^a^	11.20 ± 0.53^b^
0.05% BA + 0.05% CUR/PVP	0.558 ± 0.038^ab^	2.27 ± 0.19^c^	13.38 ± 0.66^ab^

*^*1*^Data are presented as mean ± SE from 12 chickens. P < 0.05. ^*a*-*c*^Values within columns with different superscripts differ significantly (*P* < 0.05).*

In the present study, only the administration of 0.1% CUR/PVP was able to significantly reduce the intestinal permeability of FITC-d when compared to the control non-treated chickens. However, only chickens that received CUR/PVP-BA showed significant reduction of serum SOD, while those that received CUR/PVP significantly increased serum SOD compared to the other experimental groups. Interestingly, once again, chickens that received 0.1% CUR/PVP showed a significant reduction on total intestinal IgA when compared to the control non-treated chickens (**Table 6**).

## Discussion

In the United States, foodborne infections cause more than seventy million infections and an average of five thousand loss of life each year (Allos et al., 2004) with an annual estimated cost of 51 billion dollars (Mead et al., 1999; Scharff, 2012). In addition, concerns about drug-resistant bacteria and the appearance of antimicrobial residues in animal products have driven the industry to find alternatives to antibiotic growth promoters (Subbiah, 2007; Hailu et al., 2009; Skjånes et al., 2013; Samad et al., 2016).

The *in vitro* antimicrobial activity of 1% BA against SE (**Table 4**) was similar to previously published work (Hernandez-Patlan et al., 2017). The antimicrobial activity of BA is associated with its ability to diffuse through the cell membrane of microorganisms since it is non-dissociated in the different pH of the gastrointestinal tract due to its pKa (9.1). Once inside the microorganism, BA can affect enzymatic and non-enzymatic processes such as the suppression of NADH oxidation, which decreases metabolism and leads to the subsequent death of the microorganism (Schmidt et al., 2010; Kabu and Akosman, 2013).

In the present study, the solid CUR/PVP at a 1:9 ratio (CUR/PVP) prepared and tested at 1% (10 g/Kg of feed) showed a significant reduction in SE by a 2.25 log_10_ when compared to the control non-treated group (**Table 4**). This result is in contrast with our previous results, where 1% CUR without PVP had no *in vitro* antimicrobial activity against SE. These results are justified given the increase in solubility and permeability of CUR when it is associated with PVP as displayed in the *in vitro* studies (**Tables 2, 3**). The increase in solubility might be due to the formation of soluble complexes between CUR and PVP (Kaewnopparat et al., 2009). Furthermore, the combination of 0.5% BA with 0.5% CUR/PVP confirmed the synergistic antimicrobial effect described in previous publications (Hernandez-Patlan et al., 2017).

This synergistic effect was more evident in the *in vivo* trials where administration of 0.05% CUR/PVP with 0.05% BA significantly reduced the colonization of SE in both the crop and CCT compared to the control non-treated chickens (**Table 5**). Previous studies had demonstrated the synergistic effect between CUR and BA given their interaction to form more stable complexes such as rosocyanine and rubrocurcumin (Halim et al., 2012; Wanninger et al., 2015; Hernandez-Patlan et al., 2017). These types of complexes modify the properties of CUR, which could be related to the improvement in antimicrobial activity (Priyadarsini, 2014). Interestingly, 0.1% CUR/PVP was also effective in reducing SE colonization, but this reduction occurred only in CCT in both *in vivo* experiments (**Table 5**). This is likely due to the solubility of CUR/PVP (1: 9 ratio), which is almost two times higher at pH 6.4 than at 1.2 (**Table 2**) and also due to the longer transit time in the intestine than in the crop. Several studies have shown that the solubility of CUR increases more than 25-fold at pH 6.8 when it is compared with its solubility at pH 1.2 (Song et al., 2016). It is known that at physiological pH, CUR alone rapidly degrades forming products such as bicyclopentadione, vanillin, and ferulic acid (Kharat et al., 2017).

According to our results, it is likely that the use of a solid CUR/PVP (1:9 ratio) improves the stability of CUR at alkaline pH and thus avoid its degradation. It is known that one of the most critical properties of CUR besides the antioxidant effect is its antimicrobial effect (Samarasinghe et al., 2003; Khan et al., 2012; Moghadamtousi et al., 2014). Some studies have demonstrated that the antibacterial activity of CUR is due to the inhibition of bacterial cell proliferation (Rai et al., 2008) or affecting virulence, *quorum sensing*, and biofilm initiation (Rudrappa and Bais, 2008). Nevertheless, it is clear that the antimicrobial mechanism of action of CUR is related to the membrane damage of prokaryotes (Tyagi et al., 2015). In contrast, in both *in vivo* trials, BA failed to significantly reduce SE colonization in crop and CCT (**Table 5**).

Gut integrity involves a close relationship between the microbiome, the enteric nervous system and the mucosal immunity (Gilani et al., 2016). Biological, chemical, physical, or environmental stresses can alter the gut microbiome (dysbiosis), increasing the permeability to toxins and stimulate an inflammatory response (Oz, 2017). Under healthy conditions, pathogens cannot cross the epithelial barrier, but when microbial infection occurs, pathogens can interact and damage the epithelial barrier, causing an increase in intestinal permeability (Gilani et al., 2016). Gut leakage can be measured using FITC-d as a marker of intestinal integrity (Vicuña et al., 2015b). In the present study, the group treated with CUR/PVP significantly decreased serum FITC-d levels when compared to the control non-treated group. The reduction in serum FITC-d levels could be due to the control of SE by 0.1% CUR/PVP and the capacity of CUR to increase the intestinal epithelial cell proliferation/regeneration (Bereswill et al., 2010). However, there was no reduction of serum FITC-d when 0.05% CUR/PVP is associated with 0.05% BA (CUR/PVP-BA), indicating that CUR/PVP should not be associated with BA to allow for cell regeneration.

The intestinal mucosa releases anti-microbial proteins and IgA. IgA is an antibody isotype specialized in protecting the intestinal mucosa, as well as inhibiting inflammatory processes, neutralizing bacterial toxins, and enhancing nonspecific defense mechanisms (e.g., lactoperoxidase and lactoferrin) (Gutzeit et al., 2014; Merino-Guzmán et al., 2017). Since the secretory IgA provides the first line of defense by preventing pathogen entry into the mucosa (Neutra and Kozlowski, 2006), its concentration was measured in a segment of intestine (**Table 6**). In the present study, control non-treated chickens showed the highest IgA levels. This is likely due to SE ability to induce a strong local inflammatory response in an intestinal tissue, leading to increase of pro-inflammatory cytokines secretion (Steinberg et al., 2014) which stimulates the production of IgA, antimicrobial peptides, and promote epithelial repair (Patel and McCormick, 2014). The decrease of IgA levels in the group treated with CUR/PVP is related to the anti-inflammatory properties of CUR (Gupta et al., 2012). Therefore, these results can be correlated with the low serum FITC-d levels since the mucosal inflammation process was less severe. Although there are no significant differences in the levels of IgA in the groups treated with BA, and CUR/PVP-BA, the slight decrease observed could have been because these treatments decreased the concentration of SE, as well as, the inflammatory response by regulation of enzymes (Hunt, 1996). SOD is an enzyme involved in shielding cells from the harmful reactive oxygen species (ROS) produced in response to an inflammatory process. It is known that CUR can induce the expression of cytoprotective proteins such as SOD. Furthermore, CUR is also able to react directly with ROS and reactive nitrogen species (RNS) to scavenge superoxide anions, hydroxyl radicals, H_2_O_2_, singlet oxygen, nitric oxide, peroxynitrite and peroxyl radicals (Trujillo et al., 2013; Kant et al., 2014). Chickens that received 0.1% CUR/PVP had significantly higher SOD activity. However, in the group treated with CUR/PVP-BA the activity of SOD decreased compared to the control non-treated chickens. Probably, the complexes formed between CUR/PVP and BA were not able to stimulate the expression of SOD, and it is evident that the decrease in SOD activity in this group could be due to the control of SE.

In the present study, no significant differences in SOD activity between the 0.1% BA group and control non-treated chickens were observed, although BA decreased the concentration of SE more than 1 Log_10_. Some studies have shown that boron supplementation increases the activity of SOD (Pizzorno, 2015; Bhasker et al., 2016). However, the mechanism by which the enzymatic activity is increased is not clear. Boron may also be promoting thymocyte proliferation while inhibiting apoptosis via enhancing the antioxidative function of the thymus (Jin et al., 2017).

The results suggest that the increase in the solubility and stability of CUR when it is associated with PVP could be involved in the improvement of the antimicrobial activity of CUR against SE both in the *in vitro* digestion model and in the two independent *in vivo* trials. In addition, it was confirmed in the *in vivo* trials that the combination between CUR/PVP and BA have a synergistic effect since the antimicrobial effect against SE intestinal colonization was similar to CUR/PVP but better than BA, considering that the proportion of each of them was 50% (0.05% CUR/PVP and 0.05% BA). On the other hand, 0.1% CUR/PVP was able to reduce the intestinal permeability of FITC-d and total intestinal IgA, as well as increase the activity of SOD when compared to control, while, BA-CUR/PVP only decreased SOD activity. Further studies to confirm and expand the *in vivo* results obtained in this pilot study, adding intestinal microbial commensal groups and more inflammatory biomarkers to get a complete description of the effects of BA and CUR deserves further investigation.

## Author Contributions

DH-P and GT conceived, designed, and drafted the manuscript. JL, XH-V, and GT drafted the article or revised it critically for valuable intellectual content. DH-P, BS-C, KP, JL, MB, RM-G, and AM-A acquired the data. BH, RL-A, DH-P, and GT analyzed and interpreted the data.

## Conflict of Interest Statement

The authors declare that the research was conducted in the absence of any commercial or financial relationships that could be construed as a potential conflict of interest.

## Abbreviations

BA: boric acid
BA-CUR/PVP: combination of 0.5% BA and 0.5% CUR/PVP
CCT: ceca-cecal tonsils
CUR: curcumin
CUR/PVP: dispersion of CUR with PVP
FITC-d: fluorescein isothiocyanate–dextran
PVP: polyvinylpyrrolidone
SE: solid *Salmonella enterica* serovar Enteritidis
SOD: superoxide dismutase
